# Systemic Degree of Perturbation of Plasma Markers Reveals Cumulative Biological Stress Across the Competitive Season in Professional Soccer Players

**DOI:** 10.1007/s40279-026-02470-z

**Published:** 2026-06-22

**Authors:** Deivide Oliveira-de-Souza, Diego Viana Gomes, Juliano Spineti, Antônio Pedro Farias Neto, Humberto Miranda, Bruna Karoline Lima Piazera, Ricardo Luzardo, Artur T. L. Queiroz, Beatriz Barreto-Duarte, Bruno B. Andrade

**Affiliations:** 1 Programa de Pós-Graduação em Medicina e Saúde Humana, Escola Bahiana de Medicina e Saúde Pública (EBMSP), Salvador, Bahia, Brazil; 2 Multinational Organization Network Sponsoring Translational and Epidemiological Research (MONSTER) Initiative, Salvador, Bahia, Brazil; 3 Departamento de Corridas, Escola de Educação Física e Desportos, Universidade Federal do Rio de Janeiro, Rio de Janeiro, Brazil; 4 Departamento de Fisiologia, Fluminense Football Club, Rio de Janeiro, Brazil; 5 Universidade Federal do Rio de Janeiro (UFRJ), Rio de Janeiro, Brazil; 6 Laboratório de Desempenho, Treinamento e Exercício Físico (LADTEF-UFRJ), Rio de Janeiro, Brasil; 7 Centro Universitário de Barra Mansa (UBM), Rio de Janeiro, Brazil; 8 Diretoria de Operações Acadêmicas, Clariens Educação, São Paulo, Brazil; 9 Laboratory of Clinical and Translational Research, Gonçalo Moniz Institute, Oswaldo Cruz Foundation, Salvador, Brazil; 10 Graduate Program in Biotechnology and Investigative Medicine, Gonçalo Moniz Institute, Oswaldo Cruz Foundation, Salvador, Brazil; 11 The Genios Lab, Multinational Organization Network Sponsoring Translational and Epidemiological Research (MONSTER) Initiative, Salvador, Bahia Brazil; 12 Institute for Research in Priority Populations (IRPP), Multinational Organization Network Sponsoring Translational and Epidemiological Research (MONSTER) Initiative, Salvador, Brazil; 13 Instituto de Pesquisa Clínica e Translacional, Medicina Zarns, Clariens Educação, Salvador, Brazil; 14 Division of Infectious Diseases, Johns Hopkins University School of Medicine, Baltimore, MD USA; 15 Department of International Health, Bloomberg School of Public Health, Johns Hopkins University, Baltimore, MD USA; 16 Laboratório de Pesquisa Clínica e Translacional, Instituto Gonçalo Moniz, Fundação Oswaldo Cruz, Rua Waldemar Falcão, 121, Candeal, Salvador, Bahia 40296-710 Brazil

## Abstract

**Background:**

Fixture congestion has emerged as one of the defining challenges of modern professional soccer, exposing players to repeated high-intensity demands with limited opportunities for recovery. Such cumulative exposure taxes multiple physiological systems and may lead to sustained biological strain throughout the competitive season. However, the integrated systemic impact of congested schedules remains incompletely characterized.

**Objective:**

We aimed to characterize the systemic physiological impact of cumulative competitive load across a full professional soccer season using an integrated biomarker framework.

**Methods:**

In this prospective longitudinal investigation, we employed the systemic degree of perturbation score, an adaptation of the molecular degree of perturbation, to circulating biochemical, hematological, and metabolic biomarkers obtained from professional soccer players across a full competitive season. Multi-dimensional analyses were employed to characterize systemic alterations associated with cumulative competitive load.

**Results:**

A progressive increase in systemic perturbation was observed across the competitive season. The relative contribution of individual biomarkers shifted over time, with late-season phases characterized by more integrated muscular-inflammatory-metabolic profiles. Myoglobin and monocyte-related markers consistently contributed to systemic degree of perturbation values across competitive quarters, and network analyses demonstrated progressive changes in correlation architecture involving muscular, inflammatory, and metabolic mediators.

**Conclusions:**

These results provide a comprehensive view of the physiological burden imposed by a professional soccer season and describe a progressive integration of muscular, inflammatory, and metabolic responses under cumulative competitive load. This integrated perspective supports the potential utility of biomarker-based monitoring frameworks for characterizing seasonal biological strain in elite athletes.

**Supplementary Information:**

The online version contains supplementary material available at https://doi.org/10.1007/s40279-026-02470-z.

## Key Points

**Table:** 

The systemic degree of perturbation revealed a progressive accumulation of muscular, inflammatory, and metabolic stress as the season advanced.
Myoglobin levels and monocyte counts demonstrated consistent seasonal associations with systemic degree of perturbation values, highlighting their potential as stable indicators of accumulated biological stress.
Integrated biomarker monitoring provides a practical framework to track systemic load and support recovery and load management strategies in elite soccer.

## Introduction

Fixture congestion in soccer is the issue of playing multiple matches in a short period, typically with less than 96 h of recovery time between games [1]. Such a condition has emerged as one of the most critical challenges in modern professional soccer, imposing repetitive high-intensity demands with limited opportunities for recovery. Elite players often face two or more competitive matches per week, interspersed with travel and training loads, a scenario frequently associated with heightened injury incidence and, in specific contexts, reduced performance [1–3]. In fact, elite professional clubs may be exposed to more than 20 weeks of fixture congestion within a single competitive season, while individual players can experience up to 10 consecutive weeks because of congested schedules [4, 5]. Beyond acute strain, there is growing concern that cumulative exposure may impose sustained physiological stress with long-term consequences for athlete health and career longevity [6].

Competitive soccer imposes a multi-faceted physiological load extending beyond the musculoskeletal system, triggering metabolic, endocrine, and immune responses [7–9]. Intense and repeated match exposure induces muscle strain, oxidative and metabolic stress, and transient immune activation. These processes are initially adaptive but may evolve into sustained physiological dysregulation when recovery is insufficient [10, 11]. Biomarkers such as creatine kinase (CK), lactate dehydrogenase (LDH), aspartate aminotransferase (AST) and C-reactive protein (CRP) are routinely used to track these physiological responses [12, 13], yet they capture only isolated facets of broader systemic adaptation. Most investigations have focused on short-term alterations following individual matches or training sessions, whereas longitudinal assessments across an entire competitive season remain scarce. Consequently, the integrated biological impact of prolonged competitive overload remains poorly understood.

A deeper understanding of how season-long exposure influences muscular, immune, and metabolic pathways is essential to improve evidence-based strategies for recovery, training periodization, and injury prevention. Although biochemical monitoring is common in elite soccer, current approaches assess physiological responses in isolation and fail to quantify the cumulative systemic strain imposed by a full season [14, 15]. To address this gap, we applied an integrated analytical framework that standardizes biomarker values relative to preseason reference levels and combines deviations across muscular, inflammatory, and metabolic domains into a single systemic index. This approach, previously applied in the context of infectious diseases [12, 13], enables a comprehensive assessment of the hidden physiological burden accumulated throughout the season and provides new opportunities for monitoring adaptation, recovery, and long-term athlete health.

We hypothesized that sustained competitive exposure across a congested season induces a progressive and coordinated systemic biological response involving muscular, inflammatory, and metabolic domains. The objective of this study was to longitudinally characterize the cumulative physiological impact of a full professional soccer season by integrating circulating biomarkers into a systemic perturbation framework and identifying key contributors to seasonal biological strain.

## Methods

### Ethics Statement

All procedures were conducted in accordance with the principles expressed in the Declaration of Helsinki. The study protocol was approved by the Research Ethics Committee of Centro Universitário de Volta Redonda/Fundação Oswaldo Aranha (UNIFOA; approval number 68281723.4.0000.5237). Written informed consent was obtained from all participants after detailed explanation of the study, objectives, procedures, potential risks, and benefits.

### Study Design and Participants

This prospective observational exposure-based study with repeated match assessments was conducted throughout the 2024 competitive season in professional soccer players from a single top-division club that competed in five official domestic and continental competitions during the season. This single-club design characterizes the investigation as exploratory while ensuring a consistent and standardized competitive and testing environment across the season. All participants were male outfield players who participated in at least one official first-team match for more than 30 min during the 2024 season. Blood samples were collected at two standardized timepoints: (i) at preseason baseline during team presentation at the start of the competitive calendar, and (ii) 12–18 h after official matches. To examine temporal dynamics across the season, post-match samples were stratified into four chronological quarters (Q1–Q4) based on the order of official fixtures. The competitive season spanned from January to December 2024 and was divided into four consecutive 3-month calendar intervals: Q1 (January-March), Q2 (April-June), Q3 (July–September), and Q4 (October-December). At baseline, samples were obtained from 53 individual players. The distribution of players by playing position was as follows: center-backs (*n* = 13), full-backs (*n* = 11), midfielders (*n* = 17), and forwards (*n* = 12). Throughout the season, post-match collections resulted in Q1 (*n* = 50), Q2 (*n* = 45), Q3 (*n* = 84), and Q4 (*n* = 66) player-match observations. Maximal oxygen uptake was estimated in the morning using the Yo-Yo Intermittent Recovery Test Level 1 [16, 17]. Body composition was assessed using a four-skinfold protocol: triceps, subscapular, suprailiac, abdominal. Total body mass was measured using a digital scale (Toledo, Brazil) and skinfold thickness was assessed with a caliper (Lange, USA). Body fat percentage was estimated using the Faulkner equation.

### Blood Sample Collection and Biochemical Analyses

Capillary blood was collected 12–18 h after official matches following standardized pre-analytical procedures to minimize variability. This time window was selected to capture a comparable subacute recovery phase across fixtures, ensuring temporal consistency throughout the season. Although distinct biomarkers exhibit different post-exercise kinetic profiles, strict standardization of the 12–18 h interval ensured internal comparability across competitive quarters while minimizing interference from subsequent training sessions, travel demands, or individualized recovery interventions. Therefore, differences in biomarker kinetics should be considered when interpreting comparisons across marker classes. Collections were obtained via a fingertip puncture at the distal phalanx and collected into heparinized microtubes, yielding approximately 200–500 µL per sample. All analyses were performed immediately after collection using certified point-of-care systems under manufacturer-recommended protocols with daily quality-control calibration. Albumin, aspartate aminotransferase, LDH, CK, hydroxybutyrate dehydrogenase, creatinine, uric acid, urea, glucose (GLU), total carbon dioxide (tCO_2_), calcium, phosphate, magnesium, potassium, sodium, and chloride were analyzed on the SEAMATY SD1 system (Seamaty Co., Sichuan, China). Hematological parameters were assessed using the DP-H10 analyzer (Dymind Biotech, Shenzhen, China), providing a complete blood count with differential leukocyte and platelet indices, including neutrophils (NEU), lymphocytes, and monocytes (MID). Myoglobin (Mb), CRP, and interleukin-6 were measured using the iChroma II fluorescence immunoassay system (Boditech Med, Chuncheon, South Korea).

### Data Analysis

Median values with interquartile ranges were used as measures of central tendency and dispersion. Longitudinal changes in biochemical markers across the season were evaluated using linear mixed-effects models, with quarter included as a categorical fixed effect and player modeled as a random intercept to account for repeated measurements and within-subject correlation. This framework accommodates unbalanced observations and missing data while preserving all available samples. Models were fitted using restricted maximum likelihood estimation with Satterthwaite approximation for degrees of freedom. Results are reported as estimated mean differences relative to baseline, with standard errors, 95% confidence intervals (Wald approximation), and corresponding *P*-values. To control for multiple comparisons across biomarkers and quarter contrasts, false discovery rate (FDR) correction was applied using the Benjamini–Hochberg procedure, with statistical significance defined as FDR-adjusted *P* < 0.05. Intraclass correlation coefficients were calculated to quantify between-player variability. Analyses were conducted in R using the lme4 and lmerTest packages. Continuous variables were compared using the Mann–Whitney *U* test or the Kruskal–Wallis test followed by Dunn’s multiple comparisons post hoc test, when appropriate. *P*-values were adjusted for multiple comparisons using the Holm–Bonferroni method. A hierarchical cluster analysis (Ward’s method) using Euclidean distance was performed on mean values of z-score normalized data. Cluster stability was assessed using 100 × bootstrap resampling.

A sparse partial least squares discriminant analysis was used to compare early-season and late-season profiles (Q3 vs Q1 and Q4 vs Q1) and identify multi-variate biomarker signatures associated with seasonal progression. All biomarkers were standardized (z-score normalization) prior to analysis. The optimal number of variables to retain was determined using repeated five-fold cross-validation (ten repetitions), minimizing the balanced error rate. Models with one and two latent components were compared, and the final model was selected based on cross-validated performance. Classification accuracy, balanced error rate, and the proportion of variance explained by the retained components were reported. To evaluate loading stability, bootstrap resampling (100 iterations) was performed, and 95% confidence intervals were estimated for first component loadings to assess the robustness of variable selection. Analyses were conducted in R using the mixOmics package.

Correlations between biomarkers across competitive quarters were examined using a network analysis of Spearman correlation matrices. Only correlations with FDR-adjusted *P*-values < 0.05 were included in the network visualization. Markers exhibiting similar correlation patterns were grouped according to modularity, inferring sub-networks within the global correlation structure and depicted using Fruchterman–Reingold force-directed graph drawing.

To assess the robustness of correlation patterns, complementary bootstrapped network analyses were performed (100 iterations). For each replicate, Spearman correlation coefficients were computed, and statistical significance was determined using FDR-adjusted *P*-values < 0.05. For each bootstrap iteration, binary adjacency matrices were generated to indicate the presence or absence of significant correlations and were aggregated to quantify the frequency of each association across replicates. Network density and the node degree of the overall systemic degree of perturbation (SDP) were estimated for each replicate and used for formal topological comparisons across competitive quarters using the Kruskal–Wallis test with Dunn’s multiple comparisons post hoc test. Network construction and analyses were conducted in R using the igraph package.

### Adaptation of the Systemic Degree of Perturbation (SDP) to Examine Plasma Concentrations of Biomarkers

The SDP is based on the molecular degree of perturbation method [18], which is an adaptation of the molecular distance to health framework described by Pankla et al. [19]. In the present study, instead of using gene expression data as in Prada-Medina et al. [20], we applied the model to circulating biomarkers pre-selected from routinely measured parameters in elite soccer players. Thus, the average concentration and standard deviation of each biomarker were calculated from the preseason baseline values, which served as the physiological reference for all subsequent comparisons. The SDP score of an individual biomarker was calculated as the standardized deviation of its value relative to the preseason baseline mean, divided by the corresponding baseline standard deviation. Essentially, the SDP represents how many standard deviations a given biomarker deviates from its reference value, expressing the magnitude of systemic alteration relative to preseason homeostasis. The SDP calculation framework is described in the Results section. We applied the SDP scoring system using data on 23 biomarkers measured in professional soccer players throughout the 2024 competitive season. Consistent with the original methodology, only absolute deviations exceeding ± 2 standard deviations from the baseline mean were retained for perturbation scoring. This threshold corresponds approximately to the 95% reference interval under normality assumptions and functions as a distribution-based filtering step within the SDP framework, restricting cumulative scoring to deviations that exceed the expected variability of the reference group. By limiting the contribution of minor fluctuations within the physiological reference interval, this approach prevents inflation of the cumulative perturbation score due to the mechanical summation of small deviations. The cumulative SDP score was obtained by summing the retained absolute deviations across biomarkers. Finally, to identify overall sample perturbation, a cutoff threshold was defined as the mean accumulated SDP score of the reference group plus 2 standard deviations; values exceeding this threshold were classified as perturbed. To further assess the robustness of the baseline-based standardization approach, a complementary sensitivity analysis was performed using within-athlete z-score normalization across the full competitive season. In this alternative scaling strategy, each biomarker value was standardized relative to the individual athlete’s own seasonal mean and standard deviation, thereby removing dependence on the preseason group distribution. The resulting perturbation scores were subsequently subjected to the same mixed-effects modeling framework described above.

## Results

### Characteristics of Study Participants and Competitive Exposure

Baseline characteristics of the enrolled soccer player are summarized in Table S1 of the Electronic Supplementary Material (ESM). A total of 53 professional athletes were included, comprising 13 center-backs, 11 full-backs, 17 midfielders, and 12 forwards. Age distribution was similar across positions (*p* = 0.451). Center-backs exhibited significantly higher body weight compared with full-backs, midfielders, and forwards (median [interquartile range] in kg: 86.7 [76–88.6] vs 75.6 [72–82.2] vs 74.2 [71.2–78.8] vs 78.4 [75.3–82.6]; *P* = 0.033), as well as greater height (median [interquartile range] in cm: 183 [181–187] vs 176.5 [172.8–180.8] vs 176.5 [172–179.8] vs 179 [175–181]; *P* = 0.005). Total body fat percentage and estimated maximal oxygen uptake were similar across positions (*P* = 0.771 and *P* = 0.848). Across the competitive season, match exposure varied across chronological quarters (Table S2 of the ESM). A total of 15, 21, 20, and 11 official matches were played in Q1, Q2, Q3, and Q4, respectively. The cumulative squad match exposure corresponded to 12,321 versus 17,806 versus 16,431 versus 7481 min (Q1–Q4).

### Professional Soccer Players Exhibit Progressive Muscular and Inflammatory Alterations with Late-Emerging Metabolic Changes Across the Competitive Season

**Fig. 1:**
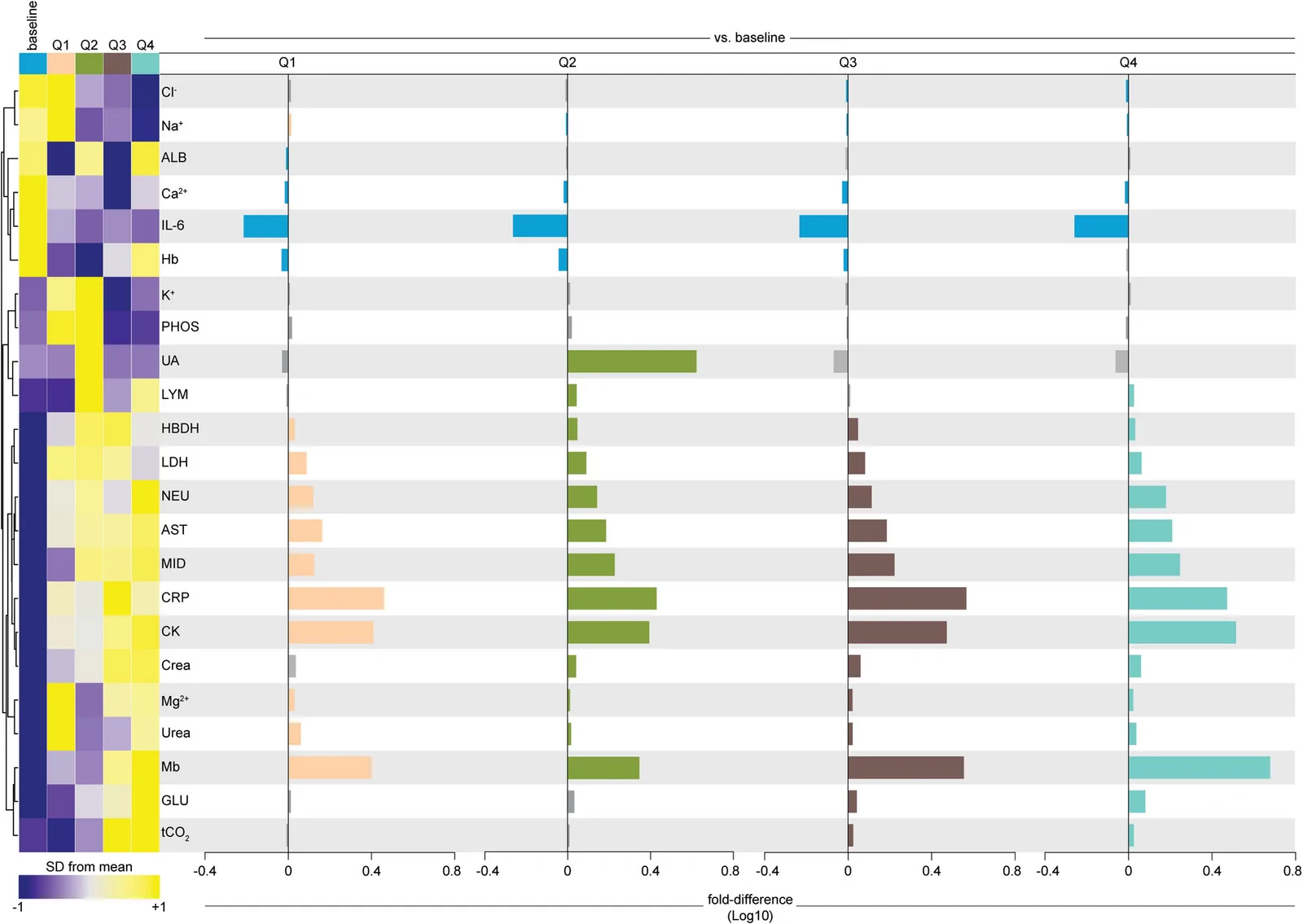
Divergence of circulating biomarker profiles from baseline across the competitive season in professional soccer players. Data were log 10 transformed and z-score normalized. A hierarchical cluster analysis (Ward method with 100 × bootstrap) was employed to test the overall expression pattern of plasma markers in the study population. Dendrograms represent Euclidean distance. The bar graphs on the *right panel* indicate the fold-change variation in marker values between the indicated timepoints. Statistical comparisons were performed using linear mixed-effects models including quarter as a fixed effect and athlete as a random intercept, with false discovery rate correction applied for multiple testing (adjusted *P* < 0.05). Significant differences are indicated by *colored bars*. *ALB* albumin, *AST* aspartate aminotransferase, *Ca*^*2*^*⁺* calcium, *CK* creatine kinase, *Cl⁻* chloride, *Crea* creatinine, *CRP* C-reactive protein, *GLU* glucose, *Hb* hemoglobin, *HBDH* hydroxybutyrate dehydrogenase, *IL-6* interleukin-6, *K⁺* potassium, *LDH* lactate dehydrogenase, *LYM* lymphocyte absolute count, *Mg*^*2*^*⁺* magnesium, *MID* mid-size cells, monocytes, *Mb* myoglobin, *Na⁺* sodium, *NEU* neutrophil absolute count, *PHOS* phosphate, *Q1* first competitive quarter, *Q2* second competitive quarter, *Q3* third competitive quarter, *Q4* fourth competitive quarter, *SD* standard deviation, *tCO₂* total carbon dioxide, *UA* uric acid

We examined the overall expression profiles of circulating markers to investigate temporal variation across the competitive season in professional soccer players (Table S3 of the ESM). Hierarchical clustering revealed progressive divergence from baseline, with Q3 and Q4 displaying the most distinct expression patterns (Fig. 1 and Table S4 of the ESM). Muscle damage markers, including CK, LDH, AST, and Mb, exhibited positive shifts beginning in Q1, with the strongest fold differences observed in Q3 and Q4. Notably, Mb exhibited the greatest magnitude of change among the assessed markers. Inflammatory mediators, such as CRP, NEU, and MID, also exhibited elevations across quarters. Hemoglobin levels showed reductions in early competitive quarters, with attenuation toward the final stage of the season. Metabolic mediators revealed a distinct temporal pattern, with GLU and tCO_2_ showing fold differences primarily in Q3 and Q4. In contrast, electrolytes demonstrated comparatively modest and less uniform changes across the season.

### Progressive Elevation of SDP Across the Competitive Season

**Fig. 2:**
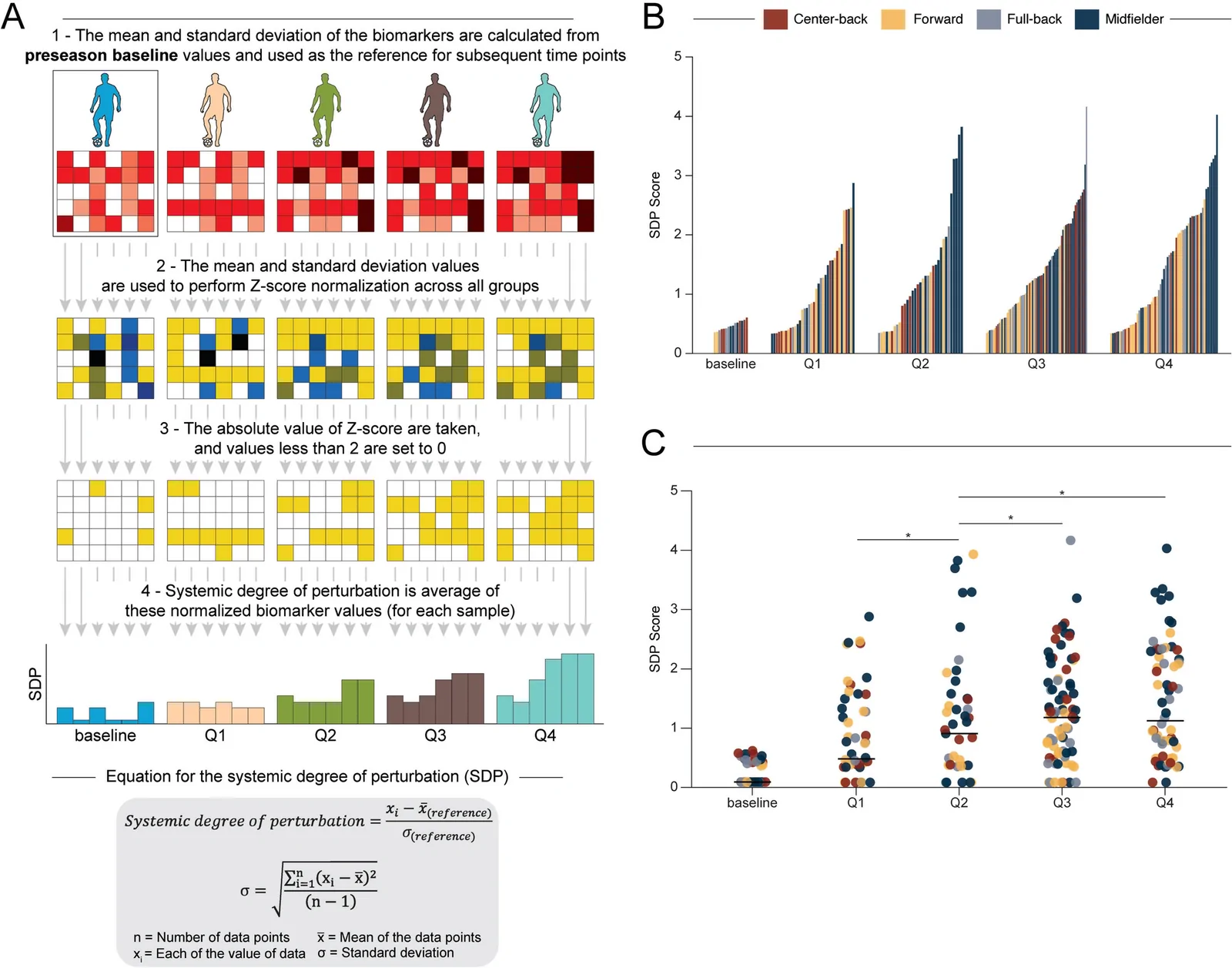
Progressive systemic degree of perturbation (SDP) across the competitive season in professional soccer players. **A** SDP is based on the molecular degree of perturbation [18], but instead of using gene expression, we used circulating biochemical markers. SDP was calculated using the mean and standard deviation of the preseason baseline group as the reference distribution. Then, z-scores were calculated for all samples, a cut-off point was established and, finally, an average disturbance calculation was performed for each sample. **B** Histogram displays the single sample SDP scores for individual players, with each bar colored according to playing position, plotted relative to the baseline group. **C** Scatter plots show SDP scores across baseline and competitive quarters (Q1–Q4). *Each dot* represents one player, with colors indicating playing position. *Horizontal lines* represent median values. Comparisons between timepoints were performed using linear mixed-effects models including quarter as a fixed effect and player as a random intercept. *P*-values were adjusted for multiple testing using false discovery rate correction. *Q1* first competitive quarter, *Q2* second competitive quarter, *Q3* third competitive quarter, *Q4* fourth competitive quarter, *P < 0.05 (adjusted)

To directly quantify the cumulative systemic impact of the competitive season, we calculated the SDP scores for each player using the framework illustrated in Fig. 2A. We found that SDP values progressively increased from baseline across the competitive season (Fig. 2B). Players exhibited significantly higher SDP scores in Q1 versus Q2 (*P* = 0.0125), Q2 versus Q3 (*P* = 0.0159), and Q2 versus Q4 (*P* = 0.0412) (Fig. 2C). Interestingly, no differences were detected between playing positions, suggesting that the progressive increase in SDP represents a collective systemic signature of seasonal progression rather than a position-specific effect.

### SDP Profiles Reveal Metabolic and Inflammatory Drivers of Late-Season Alterations

**Fig. 3:**
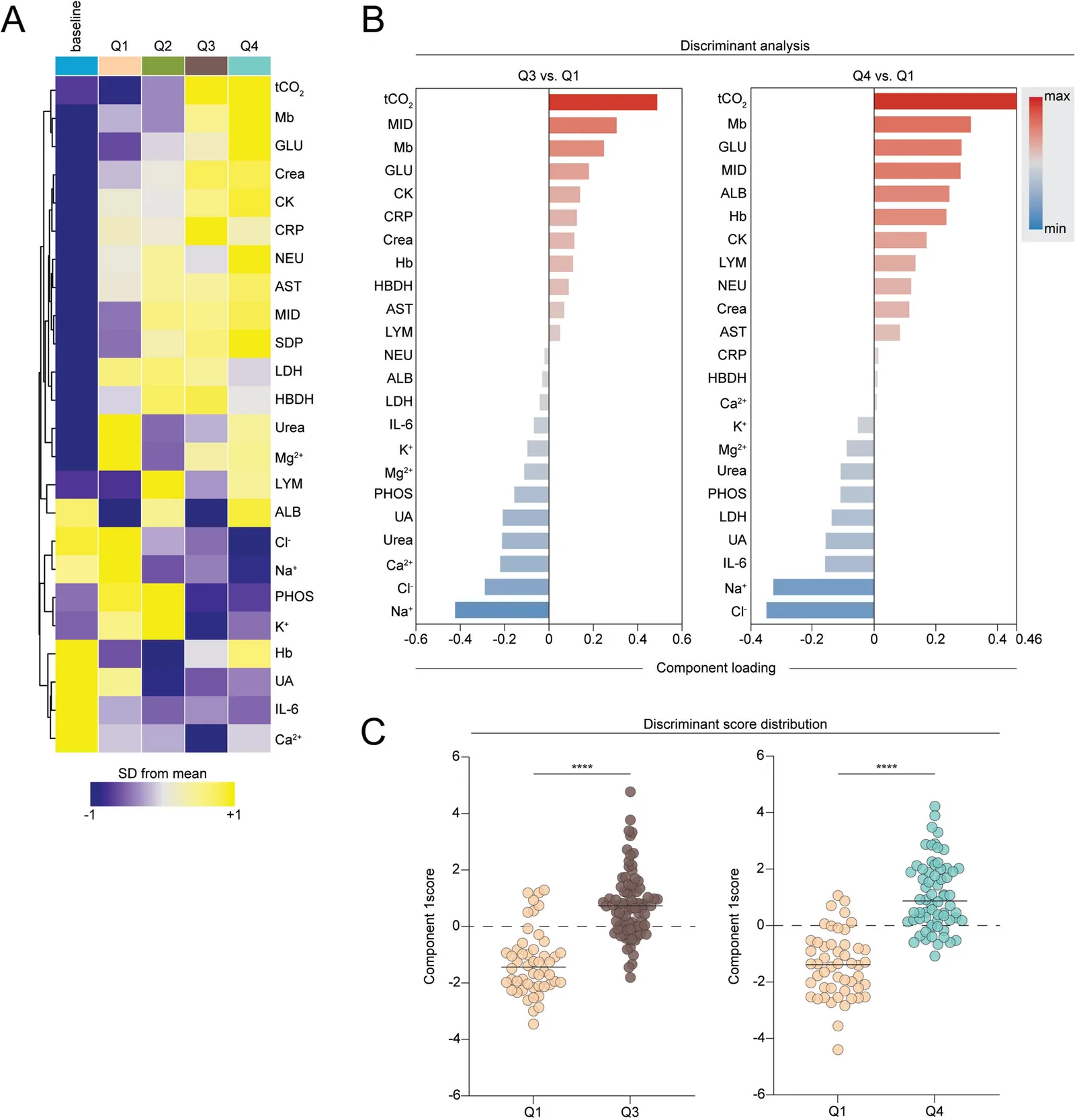
Plasma markers driving the overall systemic degree of perturbation in professional soccer players reveal distinct inflammatory profiles across competitive quarters. **A** A hierarchical cluster analysis (Ward’s method with 100 × bootstrap) using the systemic degree of perturbation values (z-score normalized) for each circulating biomarker was employed to test whether simultaneous assessment of such markers could distinguish baseline samples from those obtained during the competitive quarters (Q1–Q4) of the season. Dendrograms represent Euclidean distance. **B** A sparse partial least squares discriminant analysis model was used to identify the markers driving the discrimination between Q3 versus Q1 and Q4 versus Q1. *Bars* represent loadings of the first discriminant component. **C** Distribution of first discriminant component scores (Component 1) illustrating individual-level separation between Q1 versus Q3 (*left panel*) and Q1 versus Q4 (*right panel*). *Lines* represent median values. Differences in component scores between groups were assessed using the Mann–Whitney test. *ALB* albumin, *AST* aspartate aminotransferase, *Ca*^*2*^*⁺* calcium, *Crea* creatinine, *CK* creatine kinase, *Cl⁻* chloride, *CRP* C-reactive protein, *GLU* glucose, *Hb* hemoglobin, *HBDH* hydroxybutyrate dehydrogenase, *IL-6* interleukin-6, *K⁺* potassium, *LDH* lactate dehydrogenase, *LYM* lymphocyte absolute count, *max* maximum, *Mb* myoglobin, *Mg*^*2*^*⁺* magnesium, *MID* mid-size cells, monocytes, *min* minimum, *Na⁺* sodium, *NEU* neutrophil absolute count, *PHOS* phosphate, *Q1* first competitive quarter, *Q2* second competitive quarter, *Q3* third competitive quarter, *Q4* fourth competitive quarter, *SD* standard deviation, *tCO₂* total carbon dioxide, *UA* uric acid, ****P < 0.0001

We examined the SDP values for each plasma marker to assess whether combined perturbation profiles could distinguish baseline from competitive quarters. A hierarchical clustering analysis revealed temporal segregation between baseline and competitive states, with Q3 and Q4 displaying the most distinct perturbation profiles (Fig. 3A). In contrast, Q1 and Q2 exhibited more heterogeneous patterns, with several players clustering close to baseline. Early-season quarters were characterized by greater dispersion, while late-season states displayed a more consistent systemic signature involving muscular, inflammatory, and metabolic markers.

A discriminant analysis further supported multi-variate separation between groups (Fig. 3B), indicating that the observed differences were not explained by isolated markers but by a coordinated systemic shift. The markers with the strongest component loadings for discrimination between Q1 and Q3 were tCO₂, Mb, GLU, CK, CRP, and MID, whereas similar contributors were observed when comparing Q1 and Q4. In contrast, interleukin-6, Na⁺, Cl⁻, K⁺, and Ca^2^⁺ consistently exhibited minimal component loadings and did not substantially contribute to group separation. Of note, tCO₂ and Mb emerged as common discriminators in both late-season comparisons, indicating that these markers are central components of the perturbation profile. These findings highlight that systemic perturbation in professional soccer players is defined by integrated contributions from metabolic, muscular, and inflammatory mediators rather than by individual biomarkers. Univariate analyses of SDP values for individual biomarkers corroborated these findings (Fig. S1 of the ESM).

First discriminant component score distributions are presented in Fig. 3C. The sPLS-DA models demonstrated consistent discriminant performance in Q3 versus Q1 and Q4 versus Q1. The first discriminant component explained 11.2% and 14.5% of model variance for Q3 versus Q1 and Q4 versus Q1, respectively, with cumulative variance reaching 25.5% in the Q4 versus Q1 comparison due to retention of a second component (Table S5 of the ESM). Leave-one-out cross-validation yielded overall predictive accuracies of 85.8% and 87.1%, with cross-validated error rates below 15% in both comparisons. Similar performance was observed using repeated five-fold cross-validation (Table S5 of the ESM), supporting the stability of the discriminant models.

### Network Correlation Profiles of SDP Vary Across Competitive Quarters in Professional Soccer Players

**Fig. 4:**
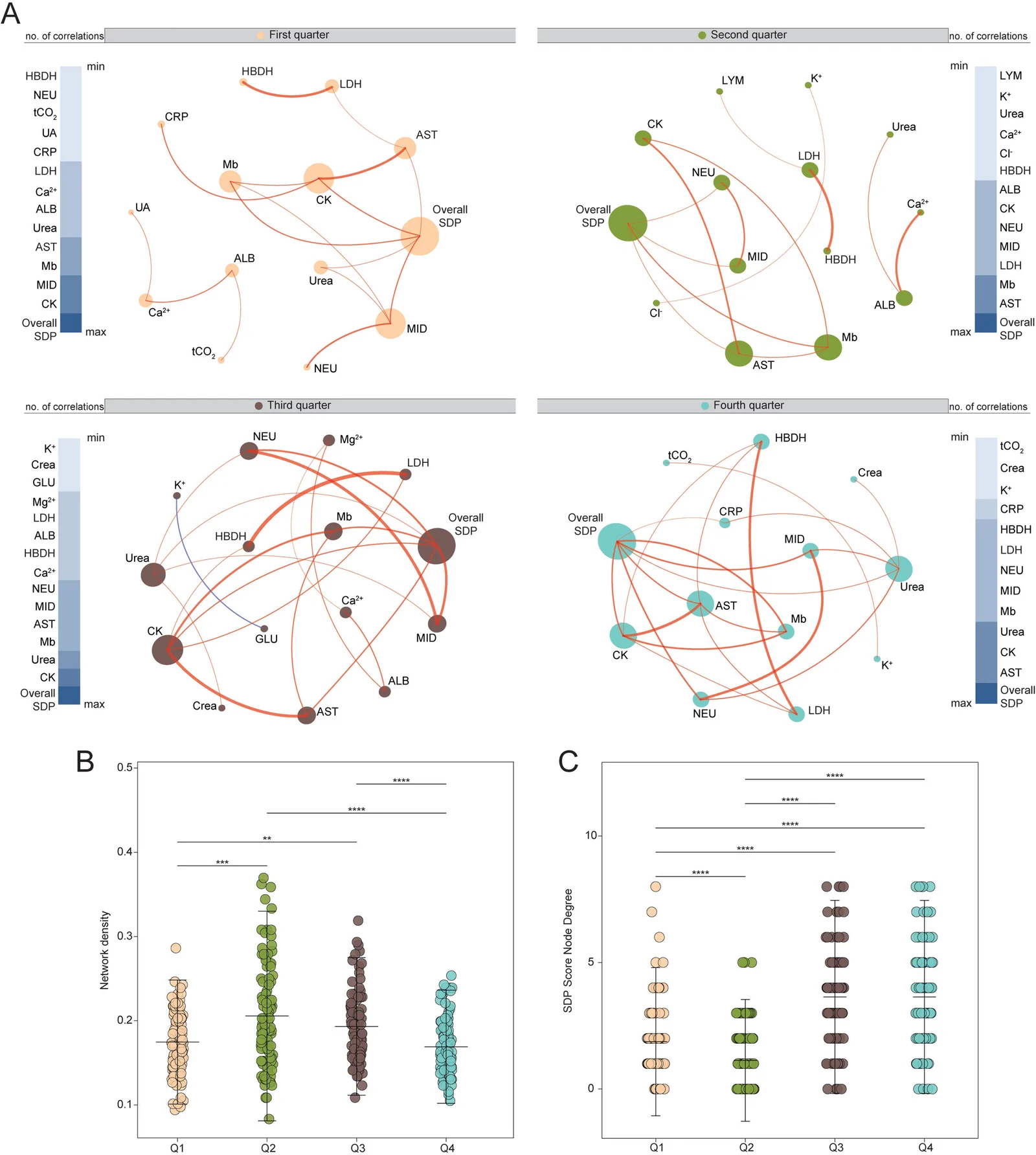
Network analysis of the systemic degree of perturbation (SDP) correlation matrices across competitive quarters. **A** Spearman correlation matrices of circulating biomarker levels in each competitive quarter were constructed, and network graphs were generated to illustrate the correlation patterns. Each *node* represents an individual biomarker, and the *node size* is proportional to the number of significant correlations observed. *Red edges* indicate positive correlations and *blue edges* indicate negative correlations. The *line thickness* reflects the correlation strength (rho value). *P*-values were adjusted for multiple testing using the false discovery rate correction, and only statistically significant correlations (adjusted *P* < 0.05) are shown. Network density **B** and node degree of the overall SDP (**C**) were estimated from 100 bootstrap iterations and compared across quarters using the Kruskal–Wallis test with Dunn’s multiple comparisons post hoc test. *Lines* represent median values. *ALB* albumin, *AST* aspartate aminotransferase, *Ca*^*2*^*⁺* calcium, *CK* creatine kinase, *Cl⁻* chloride, *Crea* creatinine, *CRP* C-reactive protein, *GLU* glucose, *Hb* hemoglobin, *HBDH* hydroxybutyrate dehydrogenase, *IL-6* interleukin-6, *K⁺* potassium, *LDH* lactate dehydrogenase, *LYM* lymphocyte absolute count, *max* maximum, *Mb* myoglobin, *Mg*^*2*^*⁺* magnesium, *MID* mid-size cells, monocytes, *min* minimum, *Na⁺* sodium, *NEU* neutrophil absolute count, *PHOS* phosphate, *Q1* first competitive quarter, *Q2* second competitive quarter, *Q3* third competitive quarter, *Q4* fourth competitive quarter, *tCO₂* total carbon dioxide, *UA* uric acid, **P < 0.01; ***P < 0.001; ****P < 0.0001 (adjusted)

To investigate the differences between SDP of individual markers and their direct effect on overall SDP values, we employed a network analysis based on Spearman correlation matrices across competitive quarters (Fig. 4). Network architecture across competitive quarters (Fig. 4A) revealed that the overall number and strength of correlations increased from Q1 to Q2, followed by relative stabilization in Q3 and reorganization in Q4, indicating structured differences in correlation patterns involving muscle damage and metabolic and inflammatory mediators. In Q1, overall SDP connected with AST, CK, Mb, urea, and MID, forming an initial core of muscle injury and metabolic stress, while other mediators such as CRP and electrolytes remained peripheral. As the season advanced in Q2, immune mediators became more prominent, with overall SDP linking to NEU, MID, Mb and AST, highlighting that systemic responses early in the season were partly shaped by inflammatory cell activity.

In Q3, the network remained highly interconnected, with overall SDP displaying broad connectivity with MID, AST, CK, Mb, urea, and NEU, indicating the convergence of muscular injury and immune activation within the correlation network. Toward the final quarter, this architecture reorganized into a more selective but stronger configuration, with the perturbation index retaining robust connections to CK, CRP, Mb, MID, urea, AST, and NEU, reflecting the consolidation of muscular and inflammatory pathways at the end of the season. Myoglobin consistently participated in these interactions across all quarters, emerging as a recurrent node within the systemic perturbation network across the competitive season. These structural changes were quantitatively supported by bootstrapped differences in network density across quarters (Fig. 4B), which peaked in Q2.

Importantly, while global network density was highest in Q2, the centrality of the overall SDP progressively increased in later quarters (Fig. 4C). These results suggest that SDP in professional soccer players is not defined by isolated changes but rather by progressive strengthening of coordinated correlation patterns among muscular, inflammatory, and metabolic mediators.

## Discussion

Physiological responses to training and competition in professional soccer have been extensively investigated, with multiple studies characterizing performance metrics, biochemical markers, and recovery strategies in elite players [2, 7, 10, 21]. The biological consequences of congested match calendars and cumulative competitive load remain poorly defined at the systemic level. This is particularly relevant given the growing debate surrounding fixture density where excessive demands are increasingly linked to injury and reduced performance [1, 2, 22]. The present study provides, to the best of our knowledge, a novel application of the SDP framework for the longitudinal quantification of the systemic biological consequences of fixture congestion across a full professional soccer season. While longitudinal biomarkers have been previously explored, our approach integrates multiple circulating signals into a single composite systemic index, offering a distinct perspective on cumulative biological strain. We employed an adaptation of the molecular degree of perturbation framework to standardize biomarker data from professional soccer players against preseason reference values, resulting in an SDP score. This metric captures the deviation of individual players from their baseline profile and condenses information from multiple circulating biomarkers into a single systemic index. The approach has been previously applied in the context of gene expression [20, 23] and immuno-inflammatory assays [24, 25]. Here, we expand its use to professional soccer. Our findings are consistent with the hypothesis that sustained competitive exposure is associated with a progressive coordinated systemic response across the season. Elevation of muscle damage markers such as CK has long been recognized as a hallmark of strenuous exercise and is routinely used to monitor recovery and injury risk in athletes [13, 26, 27]. However, the systemic implications of their sustained elevation in the context of congested match schedules remain poorly understood. In our findings, markers of muscular strain, such as CK, LDH, AST, and Mb, increased from the early stages and were accompanied by increases in CRP, NEU, and MID, indicating that immune activation accompanies cumulative muscular stress under repeated high-intensity loading with constrained recovery [28–30]. Alterations in GLU and tCO₂ became more evident later in the season, suggesting that metabolic dysregulation may represent a delayed manifestation of cumulative stress rather than an early adaptive signal. Our findings are consistent with previous studies [31, 32], which also reported progressive CK, CRP, and other muscle and inflammation-related markers during congested competitive schedules. However, unlike those studies, our analysis integrates these markers into a single systemic index, offering deeper insight into cumulative physiological strain. Using this integrative approach, we observed that professional players exhibited a progressive rise in overall SDP values across the season, reflecting the accumulation of systemic biological strain with advancing competitive load, independent of playing position. This finding reinforces ongoing concerns regarding the physiological cost of dense match schedules in elite soccer and highlights the value of integrated biomarker approaches for capturing the hidden load of competition.

Muscular stress and inflammatory activation are well-recognized consequences of repeated high-intensity exercise, implicated in performance decline [10, 11, 13, 29, 33] and, when compounded by congested schedules, greater injury susceptibility in elite athletes [1, 2, 22]. We expanded analyses to show the degree of perturbation of each individual biomarker, revealing that muscular stress and inflammatory activation were sustained hallmarks of systemic stress across the competitive season, with a more distinct perturbation profile emerging in the later stages. Markers used to index strain and recovery, notably CK and AST [12, 34–37], exhibited persistent perturbation, underscoring their utility for tracking the biological cost of professional soccer. Beyond conventional enzymes, Mb emerged as a recurrent feature across all systemic perturbation analyses, supporting its potential role as a recurrent contributor to cumulative biological strain. In contrast to CK, Mb rises promptly after sarcolemma disruption and engages mitochondrial redox pathways, linking membrane stress to systemic oxidative-metabolic imbalance [38–41]. Clinically, this faster kinetic response and its link to oxidative metabolism [42, 43] make Mb a sensitive indicator of early or subclinical muscular overload. Persistent elevation, particularly in the context of congested schedules, may reveal incomplete recovery or emerging cumulative strain even when CK remains within normal variation, providing complementary insight into the systemic consequences of competitive overload and enhancing the precision of load monitoring and recovery decisions.

In parallel, inflammatory mediators such as CRP and leukocyte subsets followed similar trajectories. While NEU responses are well established in repeated exertion [44, 45], consistent involvement of MID emerged, underscoring the relevance of this cell population, often underemphasized in athlete monitoring, despite a well-documented role in tissue repair, cytokine signaling, and coordination of recovery from eccentric stress [46, 47]. The convergence of muscular and immune markers in the systemic perturbation emphasizes that competitive overload reflects not only localized muscular stress but also an integrated muscular-immune response that intensified with seasonal progression, thereby pointing to Mb and MID as potential candidates for refined biomarker panels and warranting further exploration in athlete-monitoring frameworks. Notably, while this muscular-immune axis persisted throughout the season, metabolic dysregulation emerged later as an additional layer of systemic strain. These results support the notion that cumulative load orchestrates a coordinated muscular-immune adaptation rather than isolated tissue responses.

Metabolic regulation is essential for sustaining high-intensity efforts across congested match schedules [48] but circulating metabolic markers often receive less attention than muscular or inflammatory indicators. Here, we observed that GLU and tCO_2_ displayed consistent deviations in SDP values in the later stages of the season, contrasting with the earlier and sustained changes observed in muscle damage enzymes and inflammatory mediators. This pattern suggests that metabolic dysregulation may represent a delayed manifestation of cumulative overload. We hypothesize that such alterations could be associated with sustained neuroendocrine activation involving cortisol and catecholamines. These hormones, known regulators of GLU availability and acid–base balance during exercise [48–51], have been previously described in acute contexts. However, our findings do not allow direct determination of whether these pathways are involved in the observed late-season metabolic alterations, as hormonal concentrations were not assessed in the present study. Further investigations incorporating endocrine and expanded metabolic profiling are warranted to clarify these potential mechanisms. Monitoring metabolic markers late in the season could therefore serve as an early warning for overreaching and inform adjustments in nutritional or recovery interventions.

To capture the systemic architecture of biological stress, we extended our analyses beyond single-marker perturbation and applied discriminant analyses of SDP values. This approach revealed that separation between baseline and competitive states was not driven by isolated biomarkers but by coordinated contributions spanning muscular, inflammatory, and metabolic domains. Our findings highlight Mb, MID, and tCO_2_ as recurrent contributors, variables rarely prioritized in athlete monitoring, while interleukin-6 and electrolytes provided little discriminatory value. A key gap in the literature is whether these coordinated systemic patterns are associated with performance decrements or injury risk beyond single-analyte approaches. Our findings provide an initial step toward answering this question by demonstrating that the SDP captures the cumulative biological burden through an integrated multi-variate framework, rather than relying on isolated conventional markers.

The systemic stress response in elite athletes results from an intricate interplay between muscular, inflammatory, and metabolic domains and can be systematically characterized through a network analysis. Using this approach, we showed that the correlation profiles between SDP values of individual biomarkers and the overall SDP changed markedly across the season. Over time, the overall SDP increased its connectivity, particularly in later competitive phases, consistent with a shift toward greater systemic centralization under cumulative load. In the initial stages, overall SDP was chiefly linked to muscle-metabolic mediators such as CK, Mb, AST, and urea, consistent with early perturbations being driven by muscular strain with limited immune involvement. Importantly, global network density peaked in Q2, which corresponded to the period of highest match exposure and cumulative playing minutes during the season (Table S2 of the ESM). This phase of greater match exposure coincided with a more diffuse systemic load, simultaneously engaging muscular, metabolic, and inflammatory pathways, thereby increasing overall inter-marker connectivity within the correlation network [1, 7, 28]. As competition advanced, the network exhibited a relative reduction in global density followed by progressive reorganization, with the systemic index displaying broader connectivity to NEU and MID alongside muscle enzymes, reflecting a progressively more integrated muscular-immune-metabolic response [52, 53]. Such dissociation between peak global density in Q2 and the subsequent increase in centrality of the systemic index suggests progressive reorganization of physiological interactions under sustained competitive load, consistent with concepts in network physiology of exercise, suggesting that physiological systems reorganize their interactions under sustained stress [54]. By the final quarter, while the network exhibited relative reduction in global density, overall SDP exhibited its highest centrality, showing strong associations with CK, Mb, AST, CRP, NEU, MID, and urea. This progressive strengthening of correlations with the systemic index suggests that, as the season advances, biological responses become increasingly aligned with the overall systemic burden rather than with isolated fluctuations of single markers. Bootstrapped structural analyses confirmed that these differences reflect structured reorganization of network connectivity across competitive quarters. While network-based interpretations represent integrative frameworks rather than direct mechanistic proof, the consistency and statistical robustness of these patterns support the presence of coordinated systemic shifts under cumulative competitive load. Notably, Mb and MID remained consistently connected across stages, underscoring their potential as stable indicators of cumulative biological strain in professional soccer [41, 46].

Congested competitive calendars are consistently associated with increased injury risk and reduced performance in professional soccer [1, 2, 22]. Monitoring practices still rely heavily on isolated markers such as CK, which provide only a partial view of the biological cost of competition [13, 32, 55]. Our findings demonstrate that systemic responses to cumulative load can be quantified through SDP, an integrated metric that condenses muscular, inflammatory, and metabolic perturbation into a single systemic index. Monitoring SDP trajectories across the season may inform recovery, rotation, and return-to-play considerations, offering a more integrated physiological perspective than reliance on isolated markers. The recurrent involvement of Mb, MID, and tCO_2_ across analytical approaches indicates that these variables may serve as under-recognized indicators of cumulative strain, complementing classical markers in the monitoring of elite athletes. Furthermore, integrating SDP with external and internal load measures may improve the characterization of accumulated physiological strain, in line with recent recommendations for multi-variate monitoring in elite athletes [32, 55]. Future studies are needed to determine whether systemic perturbation profiles are associated with performance decrements or injury risk beyond current monitoring approaches.

### Limitations

This study has limitations. The relatively small and homogeneous sample of male professional soccer players from a single league may reduce the generalizability of the findings to other contexts, such as female athletes or players exposed to different competition schedules. Furthermore, we acknowledge that the observed biomarker patterns could be influenced by club-specific medical protocols, player rotation strategies, recovery practices, and local environmental conditions. While these contextual factors may limit broad generalizability, they also reflect the applied real-world nature of elite professional soccer, supporting the ecological validity of the findings. The biomarker panel was restricted to routinely available laboratory markers, meaning that additional mediators of stress and recovery, for example, hormonal or advanced inflammatory profiles, were not captured and may have provided further insight into systemic adaptation. Additional stress-related factors (e.g., training, sleep, nutrition, psychological load) were not systematically recorded, which may have contributed to unmeasured variability. Despite these constraints, the study provides novel evidence that integrated biomarker monitoring through SDP captures the cumulative biological burden of professional soccer, offering a foundation for future validation in broader cohorts. Future research should explore whether SDP trajectories predict injury risk, performance decline, or time-loss events, and whether targeted interventions based on systemic perturbation profiles can mitigate these outcomes. Expanding the biomarker panel to include hormonal, metabolic, and molecular mediators will also enhance mechanistic understanding and translational potential.

## Conclusions

This study describes a multi-systemic physiological pattern associated with cumulative competitive load in professional soccer. By integrating muscular, inflammatory, and metabolic domains into the SDP score framework, we observed coordinated systemic alterations across the season that were not fully captured by isolated biomarkers. These findings support the potential value of integrated biomarker approaches in high-demand athletic contexts and may contribute to future strategies for individualized monitoring, recovery, and load management in elite sport.

## Supplementary Information

Below is the link to the electronic supplementary material.

Supplementary file1 (PDF 8000 KB)

## Declarations

### Availability of Data and Material

The data that support the findings of this study are available from the corresponding author upon reasonable request.
