# Supplementary material for: Systemic Degree of Perturbation of Plasma Markers Reveals Cumulative Biological Stress Across the Competitive Season in Professional Soccer Players
Source: Sports Med. 2026 Jun 22;56(10):2597–610. doi: 10.1007/s40279-026-02470-z (PMC13615059; doi:10.1007/s40279-026-02470-z)
Supplement: Supplementary file 1 [file 40279_2026_2470_MOESM1_ESM.pdf]

Title: Systemic degree of perturbation of plasma markers reveals cumulative biological stress across the competitive season in professional soccer players

Journal: Sports Medicine

Authors: Deivide Oliveira-de-Souza[1,2], Diego Viana Gomes[3], Juliano Spineti[4], Antônio Pedro Farias Neto[4], Humberto Miranda[5,6], Bruna Karoline Lima Piazera[5,7], Ricardo Luzardo[8], Artur T. L. Queiroz[9,10,11], Beatriz Barreto-Duarte[2,9,12,13], Bruno B. Andrade[1,2,9,11,12,13,14,15+]

Affiliations : [1]Programa de Pós-Graduação em Medicina e Saúde Humana, Escola Bahiana de Medicina e Saúde Pública (EBMSP), Salvador, Bahia, Brazil; [2]Multinational Organization Network Sponsoring Translational and Epidemiological Research (MONSTER) Initiative, Salvador, Bahia, Brazil; [3]Departamento de Corridas, Escola de Educação Física e Desportos, Universidade Federal do Rio de Janeiro, Rio de Janeiro, Brazil; [4]Departamento de Fisiologia, Fluminense Football Club, Rio de Janeiro, Brazil; [5]Universidade Federal do Rio de Janeiro (UFRJ), Rio de Janeiro, Brazil; [6]Laboratório de Desempenho, Treinamento e Exercício Físico (LADTEF-UFRJ), Rio de Janeiro, Brasil; [7]Centro Universitário de Barra Mansa (UBM), Rio de Janeiro, Brazil; [8]Diretoria de Operações Acadêmicas, Clariens Educação, São Paulo, Brazil; [9]Laboratory of Clinical and Translational Research, Gonçalo Moniz Institute, Oswaldo Cruz Foundation, Salvador, Brazil; [10]Graduate Program in Biotechnology and Investigative Medicine, Gonçalo Moniz Institute, Oswaldo Cruz Foundation, Salvador, Brazil; [11]The Genios Lab, Multinational Organization Network Sponsoring Translational and Epidemiological Research (MONSTER) Initiative, Salvador, Bahia, Brazil; [12]Institute for Research in Priority Populations (IRPP), Multinational Organization Network Sponsoring Translational and Epidemiological Research (MONSTER) Initiative, Salvador, Brazil; [13]Instituto de Pesquisa Clínica e Translacional, Medicina Zarns, Clariens Educação, Salvador, Brazil; [14]Division of Infectious Diseases, Johns Hopkins University School of Medicine, Baltimore, Maryland, USA; [15]Department of International Health, Bloomberg School of Public Health, Johns Hopkins University, Baltimore, Maryland, USA.

Correspondence: Bruno B. Andrade, MD, PhD, Laboratório de Pesquisa e Translacional, Instituto Gonçalo Moniz, Fundação Oswaldo Cruz, Rua Waldemar Falcão, 121, Candeal, Salvador, Bahia 40296-710, Brazil. Phone: +55-71-3176-2279. E-mail: bruno.andrade@fiocruz.br

Supplemental Tables and Figures

Table S1. Characteristics of soccer player participants

Characteristic	Center Back	Full Back	Midfielder	Forward	P-value
N – no.	13	11	17	12	
Age – y	29 (22-35)	24.5 (21.8-31.2)	23 (21-28)	27 (21-30)	0.451
Weight – kg	86.7 (76-88.6)	75.6 (72-82.2)	74.2 (71.2-78.8)	78.4 (75.3-82.6)	**0.033**
Height – cm	183 (181-187)	176.5 (172.8-180.8)	176.5 (172-179.8)	179 (175-181)	**0.005**
Total Body Fat - %	11.9 (11.5-12.8)	12.8 (11.1-13.6)	11.8 (10.6-13)	11.6 (11.2-14.7)	0.771
VO_2 max - ml/kg/min	49.8 (46.8-52.1)	50.6 (49.7-53)	51.1 (49.3-53.2)	50.5 (49.5-51.9)	0.848

Data are presented as medians and interquartile ranges. Variables include age, anthropometric parameters, total body fat, and estimated VO_2 max. The Kruskal–Wallis test was used to compare distributions across playing positions. P-values in bold font are statistically significant.

Abbreviations: cm (Centimeters); kg (Kilograms); VO_2 max (Maximal Oxygen Uptake); y (Years).

Table S2. Competitive match exposure across chronological quartiles during the season

Chronological Quarter	Q1	Q2	Q3	Q4
Matches Played – no.	15	21	20	11
Total Squad Match Exposure - min	12321	17806	16431	7481

Data represent the total number of official matches played and the cumulative squad match exposure (sum of individual playing minutes across all players) within each chronological quartile (Q1-Q4) of the competitive season.

Abbreviations: min (minutes); Q1 (First competitive quarter); Q2 (Second competitive quarter); Q3 (Third competitive quarter); Q4 (Fourth competitive quarter).

Table S3. Descriptive distribution of circulating plasma markers across baseline and competitive quarters during the season

Parameter	Unit	Baseline	Q1	Q2	Q3	Q4
N		53	50	45	84	66
ALB	g/dL	4.5 (4.4 – 4.5)	4.3 (4.2 – 4.5)	4.5 (4.3 – 4.6)	4.3 (4.2 – 4.4)	4.4 (4.3 – 4.6)
AST	U/L	33 (27 – 45)	53 (39 – 76)	57 (47 – 71)	55.5 (45 – 73)	52.5 (43 – 72)
Ca^{2+}	mmol/L	2.5 (2.4 – 2.5)	2.4 (2.3 – 2.4)	2.4 (2.3 – 2.5)	2.3 (2.2 – 2.4)	2.4 (2.3 – 2.4)
Crea	mg/dL	0.9 (0.8 – 1.0)	1.0 (0.9 – 1.2)	1.0 (0.9 – 1.2)	1.0 (0.9 – 1.2)	1.1 (1.0 – 1.2)
Cl^{-}	mmol/L	105.5 (104.3 – 106.4)	105.2 (103.6 – 105.9)	104.1 (102.5 – 105.4)	103.5 (102 – 104.8)	102.8 (101.5 – 103.8)
CK	U/L	280 (176 – 395)	1167.5 (710 – 1628)	1169 (777 – 1544)	1382 (983.5 – 1776)	1312 (965 – 1964)
GLU	mg/dL	90.1 (76.8 – 98.7)	91.1 (83.9 – 103.2)	90.5 (83.6 – 102)	95.7 (89 – 110.1)	100.5 (92.7 – 124.7)
Hb	g/dL	15.6 (15 – 16.6)	14.8 (14 – 15.2)	15 (14.1 – 15.9)	15.1 (14.4 – 15.9)	15.1 (14.6 – 16.3)
HBDH	U/L	165 (141 – 198)	195.5 (173 – 226)	207 (185 – 225)	201.5 (178.5 – 230.5)	193 (179 – 209)
IL – 6	pg/mL	3.7 (3.2 – 5.3)	2.5 (2.0 – 2.8)	2.0 (2.0 – 2.5)	2.0 (2.0 – 2.9)	2.0 (2.0 – 2.4)
K^{+}	mmol/L	4.2 (3.9 – 4.5)	4.3 (4.1 – 4.5)	4.3 (4.1 – 4.5)	4.1 (3.9 – 4.4)	4.3 (3.9 – 4.4)
LDH	U/L	203 (182 – 226)	242 (211 – 274)	251.4 (228 – 265)	240 (217 – 271)	222 (210 – 247)
LYM	10^9/L	2.7 (2.5 – 2.9)	2.8 (2.3 – 3.1)	2.9 (2.5 – 3.5)	2.8 (2.3 – 3.2)	2.9 (2.5 – 3.2)
Mg^{2+}	mmol/L	1.9 (1.8 – 1.9)	2.0 (1.9 – 2.1)	2.0 (1.8 – 2.0)	2.0 (1.9 – 2.0)	2.0 (1.9 – 2.1)
MID	10^9/L	0.5 (0.4 – 0.5)	0.6 (0.4 – 0.7)	0.7 (0.6 – 0.9)	0.7 (0.5 – 1.0)	0.7 (0.6 – 0.9)
Mb	ng/mL	11.6 (8.9 – 14.9)	37 (20.4 – 48)	26.8 (18.6 – 40)	39.3 (26.8 – 61.9)	51.5 (37.2 – 72.5)
Na^{+}	mmol/L	141.1 (139.8 – 142.3)	141.9 (140.1 – 143.2)	139.5 (138.4 – 141.2)	139.4 (138.5 – 140.8)	139.2 (138 – 140.7)
NEU	10^9/L	2.8 (2.6 – 2.8)	3.4 (2.8 – 4.2)	3.3 (2.8 – 4.2)	3.1 (2.5 – 4.7)	3.8 (2.7 – 5.2)
CRP	mg/dL	0.4 (0.1 – 0.7)	1.5 (0.8 – 3.1)	1.4 (0.8 – 3.3)	1.9 (1.4 – 4.4)	2.1 (1.0 – 3.2)
PHOS	mmol/L	3.2 (2.9 – 3.6)	3.2 (3.0 – 3.6)	3.4 (3.1 – 3.7)	3.2 (2.8 – 3.5)	3.2 (3 – 3.4)
tCO_2	mmol/L	22.5 (21.3 – 23.2)	22.4 (21.9 – 23.1)	22.7 (22.2 – 23.1)	23.8 (22.9 – 24.6)	23.8 (23.4 – 24.2)
UA	mg/dL	5.1 (4.3 – 5.7)	4.5 (4.1 – 5.4)	4.3 (3.6 – 4.9)	4.2 (3.6 – 5.1)	4.3 (3.8 – 4.8)
Urea	mg/dL	34.6 (28.6 – 38.2)	38.6 (32.9 – 43.4)	34.1 (30.5 – 38.3)	34.3 (31 – 39.5)	35.7 (31.4 – 41.6)

Data represent medians and interquartile ranges for each plasma biomarker across baseline and competitive quarters (Q1 – Q4).

Abbreviations: Q1 (First competitive quarter); Q2 (Second competitive quarter); Q3 (Third competitive quarter); Q4 (Fourth competitive quarter); ALB (Albumin); AST (Aspartate Aminotransferase); Ca^{2+} (Calcium); Crea (Creatinine); Cl^{-} (Chloride); CK (Creatine Kinase); GLU (Glucose); Hb (Hemoglobin); HBDH (Hydroxybutyrate Dehydrogenase); IL – 6 (Interleukin – 6); K^{+} (Potassium); LDH (Lactate Dehydrogenase); LYM (Lymphocytes absolute count); Mg^{2+} (Magnesium); MID (Mid – size cells – monocytes); Mb (Myoglobin); Na^{+} (Sodium); NEU (Neutrophils absolute count); CRP (C – Reactive Protein); PHOS (Phosphate); tCO_2 (Total Carbon Dioxide); UA (Uric Acid); Urea (Urea).

Table S4. Estimated differences in plasma biomarker concentrations across the competitive season relative to baseline.

Parameter	Q1	Q2	Q3	Q4	P-value (vs. baseline
N	50	45	84	66	
ALB	-0.1 (-0.18 – -0.04)	0.02 (-0.05 – 0.1)	-0.1 (-0.1 – 0.0)	0.05 (-0.02 – 0.1)	**Q1: 0.0043** Q2: 0.6040 Q3: 0.0558 Q4: 0.2355
AST	18.3 (8.6 – 28)	22.7 (12.6 – 32.8)	19.4 (10.5 – 28.3)	22.7 (13.3 – 32.1)	**Q1: 0.0005 Q2: <0.0001 Q3: <0.0001 Q4: <0.0001**
Ca^{2+}	-0.1 (-0.1 – -0.04)	-0.1 (-0.15 – -0.05)	-0.15 (-0.2 – -0.1)	-0.1 (-0.1 – -0.05)	**Q1: 0.0004 Q2: 0.0001 Q3: <0.0001 Q4: 0.0001**
Crea	0.1 (0.01 – 0.2)	0.1 (0.1 – 0.2)	0.15 (0.1 – 0.2)	0.15 (0.1 – 0.2)	Q1: 0.0528 **Q2: 0.0024 Q3: 0.0003 Q4: 0.0005**
Cl^{-}	0.2 (-0.8 – 1.2)	-1.1 (-2.1 – -0.1)	-1.3 (-2.2 – -0.4)	-2.0 (-2.3 – -1.1)	Q1: 0.7521 Q2: 0.0550 **Q3: 0.0075 Q4: <0.0001**
CK	832.8 (594.1 – 1071.4)	808 (558.1 – 1058)	891.3 (668.3 – 1114.3)	1010.7 (775.7 – 1245.7)	**Q1: <0.0001 Q2: <0.0001 Q3: <0.0001 Q4: <0.0001**
GLU	2.5 (-5.8 – 10.7)	8.8 (0.2 – 17.4)	8.9 (1.4 – 16.4)	17.5 (9.5 – 25.5)	Q1: 0.6273 Q2: 0.0648 **Q3: 0.0333 Q4: <0.0001**
Hb	-1.1 (-1.9 – -0.4)	-1.5 (-2.2 – -0.7)	-0.7 (-1.4 – -0.1)	-0.3 (-0.9 – 0.4)	**Q1: 0.0035 Q2: 0.0003 Q3: 0.0431** Q4: 0.5069
HBDH	10 (-14.6 – 34.6)	21.5 (-4.2 – 47.1)	21.8 (-0.7 – 44.3)	15 (-8.75 – 38.7)	Q1: 0.5069 Q2: 0.1356 Q3: 0.0786 Q4: 0.2732
IL – 6	-1.2 (-1.5 – -0.85)	-1.3 (-1.6 – -0.9)	-1.1 (-1.4 – -0.8)	-1.2 (-1.5 – -0.8)	**Q1: <0.0001 Q2: <0.0001 Q3: <0.0001 Q4: <0.0001**
K^{+}	0.1 (-0.1 – 0.3)	0.1 (-0.1 – 0.3)	-0.02 (-0.2 – 0.2)	0.0 (-0.2 – 0.2)	Q1: 0.6040 Q2: 0.3667 Q3: 0.8739 Q4: 0.9648
LDH	42.9 (28.9 – 56.9)	48.9 (34.2 – 63.6)	43.5 (30.5 – 56.5)	31.8 (18 – 45.5)	**Q1: <0.0001 Q2: <0.0001 Q3: <0.0001 Q4: <0.0001**
LYM	-0.01 (-0.2 – 0.2)	0.25 (0.1 – 0.4)	0.05 (-0.1 – 0.2)	0.1 (-0.1 – 0.3)	Q1: 0.9329 **Q2: 0.0203** Q3: 0.6232 Q4: 0.2333
Mg^{2+}	0.1 (0.1 – 0.2)	0.1 (0.01 – 0.1)	0.1 (0.05 – 0.15)	0.1 (0.04 – 0.15)	**Q1: <0.0001 Q2: 0.0314 Q3: 0.0002 Q4: 0.0007**
MID	0.15 (0.1 – 0.2)	0.3 (0.3 – 0.4)	0.3 (0.2 – 0.35)	0.4 (0.3 – 0.4)	**Q1: <0.0001 Q2: <0.0001 Q3: <0.0001 Q4: <0.0001**

Mb	22.5<br>(12.7 – 32.3)	23.8<br>(13.5 – 34.1)	35.9<br>(26.7 – 45.1)	55<br>(45.3 – 64.6)	**Q1: <0.0001**<br>**Q2: <0.0001**<br>**Q3: <0.0001**<br>**Q4: <0.0001**
Na^+	1.1<br>(-0.01 – 2.2)	-1.3<br>(-2.5 – -0.15)	-1.1<br>(-2.1 – -0.1)	-1.7<br>(-2.8 – -0.7)	Q1: 0.0735<br>**Q2: 0.0424**<br>**Q3: 0.0461**<br>**Q4: 0.0029**
NEU	0.9<br>(0.5 – 1.3)	1.1<br>(0.7 – 1.5)	0.7<br>(0.3 – 1.1)	1.4<br>(1.1 – 1.8)	**Q1: <0.0001**<br>**Q2: <0.0001**<br>**Q3: 0.0002**<br>**Q4: <0.0001**
CRP	1.7<br>(0.1 – 2.5)	1.7<br>(0.95 – 2.5)	2.4<br>(1.7 – 3.1)	1.5<br>(0.8 – 2.3)	**Q1: <0.0001**<br>**Q2: <0.0001**<br>**Q3: <0.0001**<br>**Q4: 0.0001**
PHOS	0.1<br>(-0.1 – 0.35)	0.1<br>(-0.1 – 0.4)	-0.04<br>(-0.2 – 0.2)	-0.02<br>(-0.2 – 0.2)	Q1: 0.3400<br>Q2: 0.3055<br>Q3: 0.7790<br>Q4: 0.8739
tCO_2	-0.1<br>(-0.6 – 0.35)	0.4<br>(-0.1 – 0.8)	1.3<br>(0.85 – 1.7)	1.4<br>(0.9 – 1.8)	Q1: 0.7014<br>Q2: 0.1943<br>**Q3: <0.0001**<br>**Q4: <0.0001**
UA	-0.3<br>(-12.2 – 11.5)	16.2<br>(4.0 – 28.4)	-0.7<br>(-11.3 – 9.8)	-0.7<br>(-11.8 – 10.4)	Q1: 0.9648<br>**Q2: 0.0161**<br>Q3: 0.9314<br>Q4: 0.9329
Urea	5.2<br>(3.3 – 7.0)	4.1<br>(2.15 – 6.0)	3.6<br>(1.9 – 5.4)	5.2<br>(3.35 – 7.0)	**Q1: <0.0001**<br>**Q2: 0.0001**<br>**Q3: 0.0001**<br>**Q4: <0.0001**

Data represent estimates and 95% confidence intervals for each plasma biomarker derived from linear mixed-effects models comparing each competitive quarter (Q1–Q4) to baseline. Models included quarter as a fixed effect and player as a random intercept. P-values correspond to false discovery rate (FDR)-adjusted comparisons. P-values in bold font are statistically significant.

Abbreviations: Q1 (First competitive quarter); Q2 (Second competitive quarter); Q3 (Third competitive quarter); Q4 (Fourth competitive quarter); ALB (Albumin); AST (Aspartate Aminotransferase); Ca^{2+} (Calcium); Crea (Creatinine); Cl^- (Chloride); CK (Creatine Kinase); GLU (Glucose); Hb (Hemoglobin); HBDH (Hydroxybutyrate Dehydrogenase); IL – 6 (Interleukin – 6); K^+ (Potassium); LDH (Lactate Dehydrogenase); LYM (Lymphocytes absolute count); Mg^{2+} (Magnesium); MID (Mid – size cells – monocytes); Mb (Myoglobin); Na^+ (Sodium); NEU (Neutrophils absolute count); CRP (C – Reactive Protein); PHOS (Phosphate); tCO_2 (Total Carbon Dioxide); UA (Uric Acid); Urea (Urea).

Table S5. Cross-validation performance metrics of the sPLS-DA models comparing competitive quarters

Parameters	Q3 vs. Q1	Q4 vs. Q1
Optimal components retained	1	2
Variance explained (Comp1) - %	11.2	14.5
Cumulative variance - %	-	25.5
LOOCV error - %	14.2	12.9
LOOCV accuracy - %	85.8	87.1
5-fold error - %	18.1	15.1
5-fold accuracy - %	81.9	84.9

Data represent cross-validation performance metrics derived from sPLS-DA models, including all 23 plasma biomarkers and comparing Q3 vs Q1 and Q4 vs Q1. The Optimal components retained were determined based on tuning procedures minimizing cross-validated error rates. Variance explained corresponds to the first discriminant component (Comp1), with cumulative variance reported when applicable.

Abbreviations: Q1 (First competitive quarter); Q3 (Third competitive quarter); Q4 (Fourth competitive quarter); sPLS-DA (Sparse Partial Least Squares Discriminant Analysis); LOOCV (Leave-One-Out Cross-Validation); Comp1 (First discriminant component).

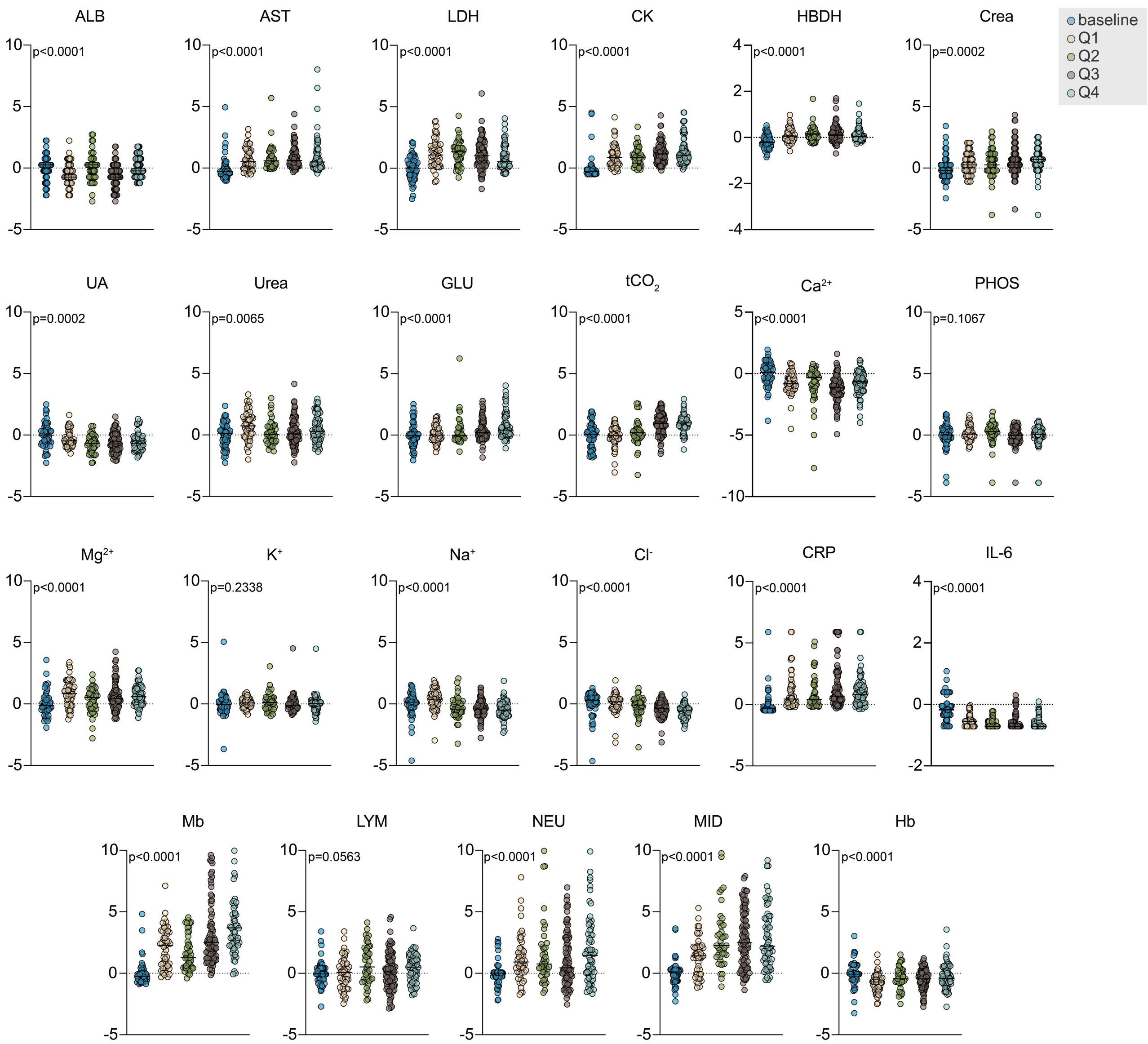

Fig. S1 Differences in the systemic perturbation of each plasma biomarker across the competitive season. Scatterplots of systemic degree of perturbation (SDP) values of the indicated biomarkers at baseline and during the competitive quarters (baseline, n=53; Q1, n=50; Q2, n=45; Q3, n=84; Q4, n=66). SDP scores are shown on the Y-axis in all plots. Lines represent median values. Formal longitudinal inference accounting for repeated measures was performed using linear mixed-effects models.

Abbreviations: Q1 (First competitive quarter); Q2 (Second competitive quarter); Q3 (Third competitive quarter); Q4 (Fourth competitive quarter); ALB (Albumin); AST (Aspartate Aminotransferase); Ca^{2+} (Calcium); Crea (Creatinine); Cl^- (Chloride); CK (Creatine Kinase); GLU (Glucose); Hb (Hemoglobin); HBDH (Hydroxybutyrate Dehydrogenase); IL – 6 (Interleukin – 6); K^+ (Potassium); LDH (Lactate Dehydrogenase); LYM (Lymphocytes absolute count); Mg^{2+} (Magnesium); MID (Mid – size cells – monocytes); Mb (Myoglobin); Na^+ (Sodium); NEU (Neutrophils absolute count); CRP (C – Reactive Protein); PHOS (Phosphate); tCO_2 (Total Carbon Dioxide); UA (Uric Acid); Urea (Urea).

STROBE Statement—checklist of items that should be included in reports of observational studies

	Item No.	Recommendation	Page No.	Relevant text from manuscript
**Title and abstract**	1	(*a*) Indicate the study's design with a commonly used term in the title or the abstract	3	Line 44
		(*b*) Provide in the abstract an informative and balanced summary of what was done and what was found	3	Lines 37-58
**Introduction**				
Background/rationale	2	Explain the scientific background and rationale for the investigation being reported	4	Lines 70-99
Objectives	3	State specific objectives, including any prespecified hypotheses	4	Lines 100-104
**Methods**				
Study design	4	Present key elements of study design early in the paper	5	Lines 113-118
Setting	5	Describe the setting, locations, and relevant dates, including periods of recruitment, exposure, follow-up, and data collection	5	Lines 113-123
Participants	6	(*a*) *Cohort study*—Give the eligibility criteria, and the sources and methods of selection of participants. Describe methods of follow-up<br>*Case-control study*—Give the eligibility criteria, and the sources and methods of case ascertainment and control selection. Give the rationale for the choice of cases and controls<br>*Cross-sectional study*—Give the eligibility criteria, and the sources and methods of selection of participants	5	Lines 116-127
		(*b*) *Cohort study*—For matched studies, give matching criteria and number of exposed and unexposed<br>*Case-control study*—For matched studies, give matching criteria and the number of controls per case	N/A	
Variables	7	Clearly define all outcomes, exposures, predictors, potential confounders, and effect modifiers. Give diagnostic criteria, if applicable	5, 6, 7	Lines 142-212
Data sources/ measurement	8*	For each variable of interest, give sources of data and details of methods of assessment (measurement). Describe comparability of assessment methods if there is more than one group	5, 6	Lines 133-150
Bias	9	Describe any efforts to address potential sources of bias	N/A	N/A
Study size	10	Explain how the study size was arrived at	5	Lines 124-127

Continued on next page

Quantitative variables	11	Explain how quantitative variables were handled in the analyses. If applicable, describe which groupings were chosen and why	6, 7	Lines 152-212
Statistical methods	12	(*a*) Describe all statistical methods, including those used to control for confounding	6, 7	Lines 152-190
		(*b*) Describe any methods used to examine subgroups and interactions	6	Lines 152-165
		(*c*) Explain how missing data were addressed	N/A	
		(*d*) *Cohort study*—If applicable, explain how loss to follow-up was addressed<br>*Case-control study*—If applicable, explain how matching of cases and controls was addressed<br>*Cross-sectional study*—If applicable, describe analytical methods taking account of sampling strategy	5	Lines 124-127
		(*e*) Describe any sensitivity analyses	6, 7	Lines 166-187
**Results**				
Participants	13*	(a) Report numbers of individuals at each stage of study—eg numbers potentially eligible, examined for eligibility, confirmed eligible, included in the study, completing follow-up, and analysed	8	Lines 215-224
		(b) Give reasons for non-participation at each stage	N/A	N/A
		(c) Consider use of a flow diagram	N/A	N/A
Descriptive data	14*	(a) Give characteristics of study participants (eg demographic, clinical, social) and information on exposures and potential confounders	8	215-221
		(b) Indicate number of participants with missing data for each variable of interest	N/A	N/A
		(c) *Cohort study*—Summarise follow-up time (eg, average and total amount)	8	Lines 222-224
Outcome data	15*	*Cohort study*—Report numbers of outcome events or summary measures over time	8, 9, 10	Lines 227-296
		Case-control study—Report numbers in each exposure category, or summary measures of exposure	N/A	N/A
		Cross-sectional study—Report numbers of outcome events or summary measures	N/A	N/A
Main results	16	(*a*) Give unadjusted estimates and, if applicable, confounder-adjusted estimates and their precision (eg, 95% confidence interval). Make clear which confounders were adjusted for and why they were included	8, 9	Lines 238-271
		(*b*) Report category boundaries when continuous variables were categorized	N/A	N/A
		(*c*) If relevant, consider translating estimates of relative risk into absolute risk for a meaningful time period	N/A	N/A

Continued on next page

Other analyses	17	Report other analyses done—eg analyses of subgroups and interactions, and sensitivity analyses	8, 9, 10	Lines 238-242; 254-271; 274-296
**Discussion**				
Key results	18	Summarise key results with reference to study objectives	11, 15	Lines 303-311; 325-330; 414-450
Limitations	19	Discuss limitations of the study, taking into account sources of potential bias or imprecision. Discuss both direction and magnitude of any potential bias	14	Lines 426-436; 437-443
Interpretation	20	Give a cautious overall interpretation of results considering objectives, limitations, multiplicity of analyses, results from similar studies, and other relevant evidence	11, 12, 13	Lines 312-330; 356-372; 383-411; 414-424
Generalisability	21	Discuss the generalisability (external validity) of the study results	13	Lines 427-443
**Other information**				
Funding	22	Give the source of funding and the role of the funders for the present study and, if applicable, for the original study on which the present article is based	16	Lines 452-453; 470-475

*Give information separately for cases and controls in case-control studies and, if applicable, for exposed and unexposed groups in cohort and cross-sectional studies.

Note: An Explanation and Elaboration article discusses each checklist item and gives methodological background and published examples of transparent reporting. The STROBE checklist is best used in conjunction with this article (freely available on the Web sites of PLoS Medicine at http://www.plosmedicine.org/, Annals of Internal Medicine at http://www.annals.org/, and Epidemiology at http://www.epidem.com/). Information on the STROBE Initiative is available at www.strobe-statement.org.
